# Differences in Short-Term Sport-Specific Functional Recovery After Primary ACL Reconstruction in the Adolescent Athlete

**DOI:** 10.1177/19417381231156395

**Published:** 2023-03-05

**Authors:** W. Craig Kemper, Connor M. Carpenter, K. John Wagner, Chien-Cheng Chen, Laura Saleem, Philip L. Wilson, Henry B. Ellis

**Affiliations:** † Texas Scottish Rite Hospital for Children, Frisco, Texas; ‡ University of Texas Southwestern Medical Center, Dallas, Texas

**Keywords:** ACL, functional testing, pediatrics, sports

## Abstract

**Background:**

Although anterior cruciate ligament (ACL) injury rates have been studied extensively, it is unclear whether levels of functional and psychological readiness for return-to-sport after primary ACL reconstruction (ACLR) differ based on an athlete’s primary sport.

**Hypothesis:**

Youth athletes in different primary sports will demonstrate differences in short-term functional recovery, as well as patient-reported psychological and functional recovery after primary ACLR.

**Study Design:**

Retrospective cohort study of consecutive patients treated for ACL injury in pediatric sports medicine clinics.

**Level of Evidence:**

Level 3.

**Methods:**

Patients included underwent primary ACLR between December 1, 2015 and December 31, 2019 and reported sports participation at the time of injury. Demographic data, sports participation, surgical data, functional testing scores (Y-Balance Test [YBT]), functional and psychological patient-reported outcome measures (PROMs), and timing of return-to-play clearance were reviewed. YBT scores were the primary metric for clearance. Four groups were studied: soccer, football, basketball, and other.

**Results:**

A total of 220 male and 223 female athletes were included; 65.28% of soccer players were female and 100% of football players were male (*P* < 0.01). At initial postoperative YBT testing (6-9 months), soccer players had higher operative (*P* < 0.01) and nonoperative (*P* < 0.01) leg composite scores when compared with basketball players. No significant differences were found between sports in functional or psychological PROMs at presurgical baseline or 6 months postoperatively. When compared with football, soccer players completed functional clearance in a shorter time from surgery (*P* = 0.02). Multivariate analysis showed level of competition as a significant independent variable for clearance in female athletes.

**Conclusion:**

After primary ACLR, athletes, especially female athletes, demonstrated short-term sport-specific differences in YBT scores. Soccer players attained clearance sooner than football players. Level of competition influenced YBT composite scores in all athletes and time to clearance in female athletes.

**Clinical Relevance:**

Sport-specific differences in reinjury should be investigated to determine whether changes in return-to-play evaluation should be implemented.

There has been an increase in incidence of anterior cruciate ligament (ACL) injury in youth athletes.^11,50^ Tear incidence has been increasing steadily in patients aged 6 to 18 years, peaking in high school.^8^ Different sports carry differing risks of ACL and lower extremity injuries. Soccer players are often cited to have an elevated risk of ACL injury.^9,43^ When analyzed by sex, ACL tear risk is highest in female soccer players and male football players.^11,43^ In a review of 5000 ACL injures in 15 sports across a collegiate-age group, football players had the greatest number of injuries. Within female sports, the highest percentage of all injuries were ACL injuries.^43^ Men’s football, women’s gymnastics, and women’s soccer had the highest rate of ACL injury per 1000 athlete exposures.^43^

When compared with adults, young patients are at increased risk of revision ACLR.^5,14,19,49,51^ Increased activity level has been highlighted as a risk factor for secondary ACL injury.^10,13,51^ Wiggins et al^51^ found that return to high levels of activity and younger age are associated with secondary ACL injury.

Due to high rates of recurrence in this age group, many have delayed return to play (RTP) from 6 months postoperatively, to 9 or 12 months and added a functional component to RTP consideration. Individualized RTP criteria have been advocated, despite minimal evidence on differences among sport-specific athletes in terms of functional or psychological recovery.

Coping after ACL injury involves various psychosocial factors. Differences in fear of reinjury, social support, and coping resources have been shown to influence recovery after primary ACLR.^3^ Fear of reinjury has also been associated with poor rehabilitation outcomes, lower return to sport rates, and higher rates of reinjury.^16,25,27,32,39^

With increased risk of secondary ACL injury in children and adolescents,^14,19^ especially those with high levels of activity,^51^ identifying other modifiable risk factors would be valuable in reducing subsequent injury in this population. The purpose of this study was to compare functional and psychological recovery after primary ACLR of adolescent athletes in 4 different populations: those who identify their primary sport as soccer, football, basketball, or other. Our hypothesis is that youth athletes in different primary sports will demonstrate differences in short-term functional recovery, as well as patient-reported psychological and functional recovery after primary ACLR.

## Methods

### Study Design

Institutional Review Board approval was obtained for the prospective collection of standardized patient-reported outcome measures (PROMs), a standardized RTP assessment, and the retrospective examination of patient data in this cohort. Consecutive records of 547 patients who underwent ACLR between December 31, 2015 and December 31, 2019 were examined.

### Participants

**Figure 1.**
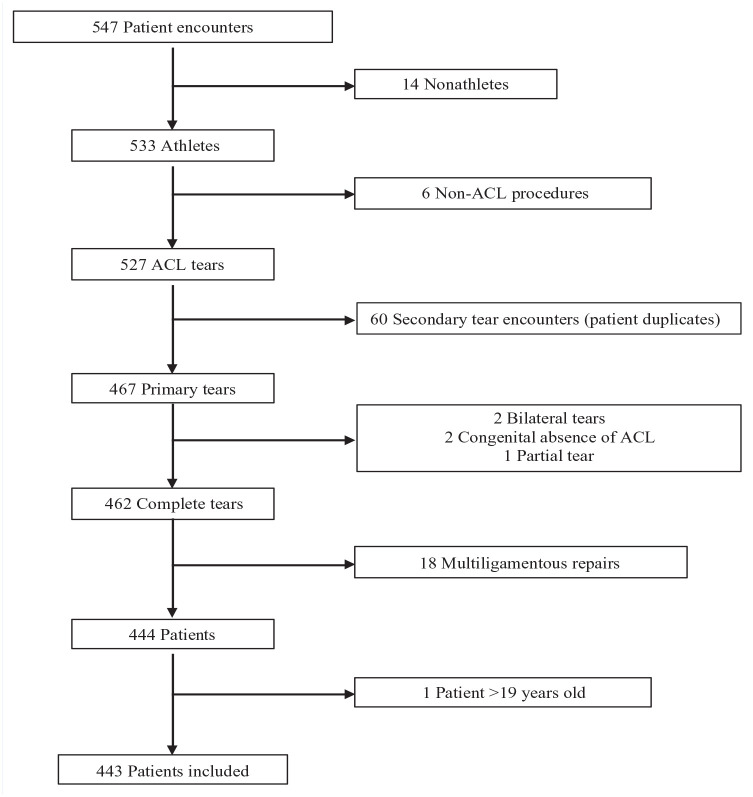
Patient inclusion. ACL, anterior cruciate ligament.

Patients were included in the study if they underwent primary ACLR within this window, were ≤19 years of age, and reported participation in sports at the time of injury. Patients were excluded if they had undergone an ACLR before December 2015, experienced bilateral or partial tears, underwent multiligamentous reconstruction/repair, or had congenital absence of an ACL. After screening, 104 patients were excluded, leaving 443 athletes for review (Figure 1).

### Demographics

Of the 443 patients, the majority identified soccer (32.51%), football (22.12%), or basketball (19.86%) as a primary sport, with the remainder classified as other (25.51%) (Table 1).

**Table 1.** Sports included

Primary Sport	Total (n = 443)
Soccer	144
Football	98
Basketball	88
Other	113
Volleyball	21
Baseball	16
Cheer	12
Track and field	12
Dance/drill team	10
Softball	9
Tennis	7
Gymnastics	6
Martial arts	4
Swimming	4
Lacrosse	3
Rugby	3
Golf	2
Wrestling	2
Motocross	2
Total	443

Participants (average age, 14.88 ± 2.25 years; range, 7-19 years) were grouped based on primary sport. The cohort consisted of 50.34% female althetes and 49.66% male althetes; 65.28% of soccer athletes were female, and 100% of football athletes were male (*P* < 0.01) (Table 2).

**Table 2.** Demographics and injury characteristics

Variable	Total (n = 443)	Soccer (n = 144)	Football (n = 98)	Basketball (n = 88)	Other (n = 113)	*P* Value^*a*^
Sex, n (%)						
Female	223 (50.34)	94 (65.28)	0 (0)	51 (57.95)	78 (69.03)	**<0.01**
Male	220 (49.66)	50 (34.72)	98 (100)	37 (42.05)	35 (30.97)	
Age at surgery, mean (range)	14.88 (7-19)	14.98 (7-19)	14.99 (9-19)	14.88 (7-18)	14.65 (7-18)	0.67
Level of competition, n (%)						
Elementary/recreational	39 (9.18)	14 (10.14)	7 (7.45)	6 (7.23)	12 (10.91)	**<0.01**
Junior high	49 (11.53)	9 (6.52)	18 (19.15)	12 (14.46)	10 (9.09)	
High school	175 (41.18)	31 (22.46)	62 (65.96)	39 (46.99)	43 (39.09)	
Select/travel	162 (38.12)	84 (60.87)	7 (7.45)	26 (31.33)	45 (40.91)	
Side of injury, n (%)						
Left	225 (50.79)	72 (50)	47 (47.96)	46 (52.27)	60 (53.10)	0.88
Right	218 (49.21)	72 (50)	51 (52.04)	42 (47.73)	53 (46.90)	
Meniscal injury, n (%)						
Lateral tear	109 (24.60)	38 (26.39)	32 (32.65)	15 (17.05)	24 (21.24)	0.16
Medial tear	86 (19.41)	28 (19.44)	13 (13.27)	16 (18.18)	29 (25.66)	
Lateral and medial tear	144 (32.51)	45 (31.25)	35 (35.71)	31 (35.23)	33 (29.20)	
No tear	104 (23.48)	33 (22.92)	18 (18.37)	26 (29.55)	27 (23.89)	

a Significant values noted in bold.

### Description of Experiment

Patient characteristics, operative data, and functional testing data were reviewed from standardized fields collected prospectively within the electronic medical records (EPIC). Y-Balance Test (YBT) data were collected at 6 to 9 months postoperatively. Functional and psychological PROMs were collected before surgery and at 6 months postsurgery.

Patients from February 2017 to December 2019 had surgical and PROM data recorded through the Outcomes Based Electronic Research Database (OBERD; Universal Research Solution LLC). A previously published validity study in pediatric patients revealed that patients often preferred OBERD to paper questionnaires.^18,44^

### Variables, Outcome Measures, Data Sources, and Bias

The following functional and psychological PROMs to assess RTP readiness were administered prospectively to all patients:

The Pediatric International Knee Documentation Committee (Pedi-IKDC) form is an adaptation of the International Knee Documentation Committee Form that has been altered and validated for use in children and adolescents to assess deterioration or improvement in symptoms.^28,31^

The Hospital for Special Surgery Pediatric Functional Activity Brief Scale is an 8-item questionnaire to assess activity level and post-treatment outcomes in patients aged 10 to 18 years.^17^

The Anterior Cruciate Ligament-Return to Sports after Injury Scale is a 12-item scale used to assess 3 psychological responses after athletic injury: emotions (5 items), confidence in performance (5 items), and risk appraisal (2 items).^48^

Previous studies have demonstrated a connection between the ability to cope and athletic injury.^24,29,30,52^ The Athletic Coping Skills Inventory-28 (ACSI-28) assesses an athlete’s ability to cope through the use of 28 questions divided into 7 subscales.^16,47^

The Athletic Identity Measurement Scale assesses athletic identity and how it affects self-concepts.^12^ Patients rate 10 statements as they relate to self-perceptions about being an athlete.^12,16,34^ Higher scores have been associated with greater rehabilitation adherence.^7,16,26^

### RTP Clearance Evaluation

Patients were evaluated for RTP no sooner than 6 months postsurgery. Functional success was assessed by certified YBT providers on the physical therapy staff. Surgeons used functional scores on YBT as the primary metric for RTP. Specific criteria for RTP include passing functional score on YBT, no effusion, pain-free arc of motion, and stable knee examination. Date of clearance was reviewed to determine time from surgery to release to activity.

A key component for evaluation of functional readiness for clearance was the YBT. In this test, the patient stands on the test kit while balancing on 1 leg. The patient slides a target marker in the anterior, posteromedial, and posterolateral directions with the other leg.^40^ Six practice attempts are performed in each direction followed by the formal test, which records the furthest successful attempt out of 3. Distance of movement is used to assess lower extremity stability for both the operative and nonoperative legs. Scores assess injury risk and movement ability.^21,35,40,41,46^ A composite score is calculated for each leg to standardize scores. This score is calculated with the following formula: [(Anterior Reach + Posteromedial Reach + Posterolateral Reach)/(3 × Limb Length)] × 100%. Patients were required to have an anterior side-to-side difference <4 cm, posteromedial and posterolateral side-to-side differences <6 cm, and a composite score >94% on both legs to record a passing score. Patients who did not pass according to these criteria underwent repeat testing 6 to 12 weeks later.

Measurements for operative and nonoperative legs were recorded in all 3 directions. Side-to-side differences were recorded for each direction. A composite score was calculated for each leg. Number of days between surgery and YBT was recorded to assess differences between athletes in recovery time before testing.

Selection bias was mitigated by selecting consecutive patients within the specified time period. Confounding from potential demographic factors such as age, gender, and activity level were addressed with multivariate analysis.

### Statistical Analysis

Categorical variables such as demographic characteristics, presence of meniscal injury, and level of competition were analyzed with descriptive statistics. These variables were compared between groups by count and percentage with Chi-square or Fisher exact tests.

Continuous variables were first examined for normality with the Shapiro-Wilk test, and nonparametric tests such as Kruskal-Wallis were considered. Continuous variables such as weight, body mass index, days between timepoints, and the various test scores were analyzed with descriptive statistics through a Kruskal-Wallis test. These variables were compared by mean, standard deviation, median, and range. Dates were recorded to track time from injury to surgery, time from injury to clearance, time from surgery to first functional test, and time from surgery to clearance. Functional scores were analyzed at 6-month timepoints, while PROMs were recorded before surgery (baseline) and at 6 months postoperatively. Average change in 6 month and baseline scores was also recorded (delta) for all PROMs.

Continuous variables that demonstrated significance were further analyzed for multiple comparisons using the Dwass, Steel, Critchlow-Fligner (DSCF) method.

Depending on the nature of outcomes, we ran ordinary regression or logistic regression analysis using age, gender, primary sport, and activity level as covariates. We also ran the same analysis, splitting the cohort into males and females.

## Results

Functional recovery was assessed using YBT scores. Athletes demonstrated differences in composite, posteromedial, and posterolateral scores. Of the 443 patients, 385 (86.91%) returned for functional testing after surgery. Average time between surgery and date of YBT was 214.79 ± 55.14 days. No difference was found between sports in average number of days from surgery to YBT (*P* = 0.40). Basketball players had lower operative leg composite scores when compared with soccer (95 ± 6.45 vs 97.83 ± 7.25, *P* < 0.01); they also posted lower nonoperative leg composite scores when compared with both soccer (96.2 ± 5.82 vs 99.41 ± 7.51, *P* < 0.01) and other (96.2 ± 5.82 vs 99.01 ± 7.2, *P* = 0.02). Soccer players posted lower average posteromedial side-to-side differences when compared with football players (3.19 ± 6.51 vs 3.68 ± 2.97, *P* = 0.03). The differences in YBT performance are summarized in Table 3. Pass rates on the 6-month YBT were highest in soccer (45.04%) (*P* < 0.01) (Table 4). Using a multivariate model, level of competition was a significant independent variable to composite operative leg score and composite nonoperative leg score. Primary sport was a significant independent variable for composite nonoperative leg score in the entire cohort and female althetes alone, but not in male althetes alone. Multivariate analysis prompted an investigation into the composite nonoperative scores of female athletes alone. Female soccer players scored higher than female basketball players (99.64 ± 6.11 vs 96.36 ± 5.31, *P* < 0.01).

**Table 3.** Mean YBT scores

Variables	Soccer (n = 131)	Football (n = 84)	Basketball (n = 75)	Other (n = 95)	*P* Value^*a*^
Anterior					
Anterior operative leg	58.04	59.79	58.64	57.73	0.27
Anterior nonoperative leg	60.24	62.00	60.97	60.38	0.19
Anterior side-to-side difference	2.95	3.52	3.05	3.33	0.36
Posteromedial					
Posteromedial operative leg	96.60	100.39	97.63	96.35	**<0.01**
Posteromedial nonoperative leg	97.01	101.71	98.29	97.42	**<0.01**
Posteromedial side-to-side difference	3.19	3.68	3.49	3.24	**0.03**
Posterolateral					
Posterolateral operative leg	93.48	96.21	94.39	92.52	**0.03**
Posterolateral nonoperative leg	94.90	97.44	94.96	94.13	**0.04**
Posterolateral side-to-side difference	3.37	3.99	3.56	3.25	0.20
Composite					
Composite operative leg	97.83	94.93	95.00	96.86	**<0.01**
Composite nonoperative leg	99.41	96.75	96.20	99.01	**<0.01**
Composite side-to-side difference	2.98	3.08	2.81	2.90	0.74

YBT, Y-Balance Test.

a Significant values noted in bold. Contiguous variables represented as means.

**Table 4.** YBT mean passage percentage

Variables	Soccer (n = 131)	Football (n = 84)	Basketball (n = 75)	Other(n = 95)	*P* value^*a*^
YBT success, n (%)					
Pass	59 (45.04)	21 (25)	22 (29.33)	42 (44.21)	**<0.01**
Fail	72 (54.96)	63 (75)	53 (70.67)	53 (55.79)	

YBT, Y-Balance Test.

a Significant value noted in bold.

No significant differences were found between sports in functional or psychological PROMs at baseline (before surgery) or 6 months. The ACSI-28 Coachability Score was the only score that showed differences in the delta between baseline and 6 months between sports (*P* < 0.01). Football players posted a greater increase in coachability score from baseline to 6 months when compared with basketball (0.86 vs -0.57; *P* < 0.01) and other (0.86 vs -0.56; *P* < 0.01). Soccer players posted significant increases in coachability score when compared with other (0.28 vs -0.56; *P* = 0.03) (Table 5).

**Table 5.** Baseline, 6-month, and delta for functional and psychological PROMs

	Soccer	Football	Basketball	Other	*P* Value^*a*^
**Baseline scores**					
Pedi-FABS total	20.86	21.30	19.87	20.61	0.51
Pedi-IKDC total	51.93	54.59	49.84	48.61	0.27
ACL-RSI	61.34	56.19	57.64	51.86	0.50
ACSI-28 coachability	10.13	9.77	10.33	10.58	0.08
ACSI-28 concentration	8.50	8.92	8.10	8.40	0.31
ACSI-28 confidence and achievement motivation	9.42	9.97	9.56	9.52	0.52
ACSI-28 coping with adversity	8.04	7.64	7.13	7.72	0.19
ACSI-28 freedom from worry	7.21	7.12	6.35	7.14	0.37
ACSI-28 goal setting and mental preparation	6.81	7.32	6.63	7.24	0.50
ACSI-28 peaking under pressure	7.96	8.24	7.39	7.39	0.22
ACSI-28 total	56.89	56.89	55.36	58.00	0.44
AIMS total	51.59	53.51	52.00	51.64	0.88
**6-month scores**					
Pedi-FABS total	17.13	17.90	17.26	16.48	0.67
Pedi-IKDC total	86.80	88.78	88.49	85.16	0.10
ACL-RSI	70.38	73.74	67.33	70.76	0.80
ACSI-28 coachability	10.55	10.44	9.72	10.11	0.23
ACSI-28 concentration	8.60	8.88	8.46	8.90	0.77
ACSI-28 confidence and achievement motivation	9.43	9.62	9.51	9.10	0.75
ACSI-28 coping with adversity	8.62	8.41	7.89	8.21	0.31
ACSI-28 freedom from worry	8.13	8.15	7.19	7.61	0.35
ACSI-28 goal setting and mental preparation	7.58	7.90	7.00	7.40	0.28
ACSI-28 peaking under pressure	8.14	8.54	7.75	7.65	0.23
ACSI-28 total	60.19	62.33	56.86	58.13	0.18
AIMS total	47.53	47.96	52.23	49.17	0.43
**Delta between baseline and 6-month scores** ^*b*^					
Pedi-FABS total	−5.26	−3.13	−2.66	−6.30	0.42
Pedi-IKDC total	37.01	30.89	40.35	35.22	0.20
ACL-RSI	9.79	10.15	16.01	15.02	0.75
ACSI-28 coachability	0.28	0.86	−0.57	−0.56	**<0.01**
ACSI-28 concentration	0.09	−0.19	−0.21	−0.05	0.89
ACSI-28 confidence and achievement motivation	0.05	−0.17	−0.63	−0.37	0.49
ACSI-28 coping with adversity	0.26	0.71	0.43	0.03	0.63
ACSI-28 freedom from worry	0.75	0.98	0.57	0.92	0.84
ACSI-28 goal setting and mental preparation	0.75	0.29	-0.14	0.71	0.35
ACSI-28 peaking under pressure	0.26	0.43	0.29	0.24	0.99
ACSI-28 total	3.20	5.00	0.18	1.08	0.56
AIMS total	−4.08	−7.26	−1.33	−2.60	0.42

ACL-RSI, Anterior Cruciate Ligament-Return to Sports after Injury Scale; ACSI-28, Athletic Coping Skills Inventory-28; AIMS, Athletic Identity Measurement Scale; Pedi-FABS, Pediatric Functional Activity Brief Scale; Pedi-IKDC, Pediatric International Knee Documentation Committee; PROMs, patient-reported outcome measures.

a Significant values noted in bold.

b Delta calculated as follows: (6-month score - baseline score). Contiguous variables represented as means.

Patients with clearance data (68.17%) indicated sport-specific differences in time from surgery to clearance (*P* = 0.03). Soccer players were cleared in fewer days after surgery than football players (285.73 ± 116.98 vs 329.53 ± 115.57; *P* = 0.02). Basketball (88.1%) and soccer (86.42%) had the highest return to same sport percentage, while football (60.53%) had the lowest percentage (*P* < 0.01) (Table 6). Using a multivariate model, level of competition was a significant independent variable to days from surgery to clearance in the entire cohort and female athletes alone, but not male athletes alone. It also showed that being a male football player was a significant factor that increased time from surgery to clearance when compared with male soccer players (*P* = 0.02).

**Table 6.** Clearance and RTP

Variables	Soccer	Football	Basketball	Other	*P* Value^*a*^
Clearance (n = 302)					
Time from surgery to clearance	285.73 ± 116.98	329.53 ± 115.57	299.18 ± 120	293.91 ± 98.57	**0.03**
Return to sport (n = 218), n (%)					
Return	70 (86.42)	23 (60.53)	37 (88.10)	41 (71.93)	**<0.01**
No return	11 (13.58)	15 (39.47)	5 (11.9)	16 (28.07)	

RTP, return to play.

a Significant values noted in bold.

## Discussion

This study examined differences in short-term recovery between pediatric athletes in different sports after primary ACLR. While broad differences were noted in select functional YBT scores after surgery, most short-term functional and psychological recovery PROMs were equivalent, with the exception of a demonstrated increase in ACSI-28 Coachability score in football and soccer players. We also found a significant positive correlation with higher level of play and earlier clearance to return to sport within female athletes. With these findings, we accept our hypothesis that there are sport-specific differences in functional recovery, yet reject our hypothesis that there are differences in short-term PROMs measuring self-reported functional and psychological recovery.

Astur et al^6^ demonstrated that skeletally immature patients who suffered ACL reruptures had lower Tegner and Lysholm functional scores upon return to activity than patients who did not suffer rerupture. This is concerning for patients who obtain lower YBT functional scores, as the YBT can also be used to assess functional readiness. Although our study did not investigate differences in secondary injury between sports, it demonstrated differences in YBT scores between sports. Because no difference was found between sports in days from surgery to YBT, any differences in YBT score were due to patient-related factors including quality of recovery, motivation, or sports-specific training that occurs during therapy, rather than time of recovery. Differences in YBT scores between sports may illustrate increased risk of subsequent injury in patients who play sports with lower averages on functional tests.

Multiple studies have demonstrated an increased risk of ACL tears in women.^4,10,15,20,23,36,42^ A meta-analysis evaluating the risk of contralateral ACL injury in women after primary ACL injury found those ≤18 years of age, those with a family history of ACL injury, those with high level of activity upon return, and those with shorter time between injury and reconstruction were at increased risk.^13^ Sex as a factor in ACL recovery is further highlighted by the results of our multivariate analysis, which showed primary sport as a significant determinant of performance on YBT composite nonoperative leg scores in female athletes, but not male athletes.

Plisky et al^41^ found that those with an anterior side-to-side difference >4 cm on the YBT were at a 2.5 times increased risk of injury to the lower extremity. In the current study, although anterior side-to-side differences did not differ significantly between sports, soccer players posted the lowest posteromedial side-to-side differences and highest composite scores. These findings, along with the shorter time from surgery to clearance for soccer when compared with football, and overall higher pass rates on YBT, suggest soccer players may functionally recover faster than football athletes. However, the risk reduction of this apparent improved functional recovery must be considered carefully in the setting of overall higher rates of ACL injury in soccer players.

PROMs are under investigation as correlates of readiness for return to activity. PROMs measured in this study included both functional and psychological tests. In this study, primary sport was not shown to be a major predictor of short-term PROMs, making it difficult to establish any link between primary sport and PROMs in this study. The only value that found significance was the calculated delta in the ACSI-28 Coachability score, which is 1 of 7 components of the overall ACSI-28 score.

Earlier time to clearance in soccer players is concerning when considering the ACL injury risk in soccer players overall.^1,22^ Wiggins et al^51^ and Cronström et al^13^ demonstrated increased risk of secondary injury in young patients returning to high level of activity. Neither study found an association between soccer participation and risk of secondary injury.^13,51^ However, soccer players in the Swedish National Knee Ligament Registry revealed that those who returned to soccer after ACLR had a significantly higher risk of sustaining further ACL injury than those who did not return.^45^ Webster et al^49^ found adolescent patients who return to cutting/pivoting sports increased the risk of graft rupture by a factor of 3.9 and contralateral injury by 5. Despite high YBT composite scores and decreased time to clearance, risk for secondary injury in soccer players calls for further investigations into other methods of RTP determination within this group to mitigate future injury risk. Nagelli et al^37^ found higher risks of secondary ACL injury in young patients and those who return within 1 versus 2 years. These findings suggest that despite higher functional scores, time as a factor in recovery should be considered, especially in populations of soccer athletes with high rates of ACL injury.

Of the soccer players included in this study, 60.87% competed at what was classified as a select/club level–a higher percentage than the other sports. Ardern et al and Lai et al^2,33^ found elite athletes more likely to return to presurgery level of play than those at lower levels. It is unclear whether preinjury physical skill-set, competitive drive, athletic identity, or additional factors are determinates of a higher rate of return to same-level of play in elite athletes. Multivariate analysis in our study revealed higher functional scores in both male and female athletes at higher levels of sport and earlier clearance in female athletes at higher levels of sport. In a study of 78 athletes aged 18 to 51 years (mean age, 31.5 years), identification as a soccer player was an independent predictor of return to sport (OR, 11.4; 95% CI, 1.12-110.64; *P* = 0.04).^38^ In the current study, there was a high number of select/club patients in the soccer group. As such, both level of play and sport may have contributed to the higher functional scores and earlier clearance for soccer players in our results.

With the findings of soccer players scoring consistently better on YBT exams and sport-specific differences in time to clearance, it is becoming evident that secondary ACL injury comparisons between sports should be conducted. Sport-specific adjustments to recovery and clearance protocols to mitigate risk of reinjury would be of great benefit to this population.

Limitations to this study include lack of uniformity in follow-up for confirmation of clearance and delay in follow-up due to COVID-19. However, days between surgery and YBT were recorded for each patient with no significant difference in these values between the sports. Clearance was defined as passing the YBT, but there were a few participants who failed the YBT but were cleared at 6 months (5.7% of 384 patients with YBT data) and a few participants who passed the YBT but were not cleared (0.7% of 384 patients with YBT data). Patients did not complete a baseline YBT before surgery. There is concern that higher postoperative scores in soccer players may be due to a higher baseline rather than faster recovery after ACLR. PROMs in this cohort were voluntary, which may have impacted follow-up percentages. Long-term (12-month and 24-month) follow-up will provide improved data regarding sport-specific RTP and PROMs.

## Conclusion

After primary ACLR, athletes in different sports demonstrate differences in YBT functional testing scores, but not in presurgery or short-term functional or psychological PROMs. This is especially true in female athletes. Differences in time from surgery to clearance were also demonstrated between sports, with soccer players attaining clearance sooner than football players. Level of competition was shown to influence YBT composite scores in male and female athletes and time to clearance in female athletes. Sport-specific differences in reinjury should be investigated to determine whether changes in evaluation for RTP should be implemented.
